# Prevalence and factors associated with timely initiation of breastfeeding in Kilimanjaro region, northern Tanzania: a cross-sectional study

**DOI:** 10.1186/s12884-020-03209-y

**Published:** 2020-09-01

**Authors:** Frank Kiwango, Innocent B. Mboya, Beatrice John, Tamara Hashim, Sia E. Msuya, Melina Mgongo

**Affiliations:** 1 Community Health Department, Institute of Public Health, Kilimanjaro Christian Medical University College (KCMUCo), P. O. Box 2240, Moshi, Tanzania; 2 Department of Epidemiology and Biostatistics, Institute of Public Health, Kilimanjaro Christian Medical University College (KCMUCo), P. O. Box 2240, Moshi, Tanzania; 3 School of Mathematics, Statistics & Computer Science, University of KwaZulu Natal, Pietermaritzburg, Private Bag X01, Scottsville, 3209 South Africa; 4 Better Health for African Mother and Child, P. O. Box 8418, Moshi, Tanzania; 5 Institute of Basic Medical Sciences, Faculty of Medicine, University of Oslo, Oslo, Norway; 6 Institute of Clinical Medicine, University of Oslo, Oslo, Norway

**Keywords:** Breastfeeding, Timely initiation, Kilimanjaro, Tanzania

## Abstract

**Background:**

The World Health Organization (WHO) recommends early initiation of breastfeeding within 1 h as it confers many benefits to the child and prevents neonatal mortality. This study aimed to determine the prevalence and factors associated with timely initiation of breastfeeding in the Kilimanjaro region, northern Tanzania.

**Methods:**

We analyzed secondary data for 866 participants from a population-based cross-sectional study conducted in April 2016 among mothers with children aged less than 5 years in three districts; Rombo, Same, and Moshi Municipal council in Kilimanjaro region, northern Tanzania. A multistage sampling selected study participants and interviewed using a questionnaire. The generalized linear model, with Poisson family and log-link function was used to estimate the prevalence ratios (PR) and 95% confidence intervals (CI) for factors associated with timely initiation of breastfeeding.

**Results:**

The prevalence of timely initiation of breastfeeding was 71.1%. The vast majority of mothers (90.7%) gave colostrum, and less than a tenth (6.4%) gave pre-lacteal feed to their children. Adjusted for other factors, not giving children prelacteal feeds remained was significantly associated with a higher prevalence of timely initiation of breastfeeding (PR: 2.22, 95%CI 1.38, 3.56, *p* = 0.001). There was no significant association between other characteristics and the likelihood of timely initiation of breastfeeding in this study.

**Conclusion:**

The prevalence of timely initiation of breastfeeding in the Kilimanjaro region was higher than the national estimate. The practice of not giving infants prelacteal feeds increased the likelihood of timely initiation of breastfeeding. There is a need to encourage mothers on the significance of recommended ANC visits and early initiation of breastfeeding to their infants to improve the practice.

## Background

Timely initiation of breastfeeding is the provision of a mother’s breast milk to infants within 1 h of birth [1]. The practice of early initiation helps the child get colostrum, which contains antibodies that help protect the infant from common childhood illnesses. Timely/ early initiation of breastfeeding promotes bonding and helps milk production [2]. Literature shows that early initiation of breastfeeding is associated with a 42% reduction in mortality rate among low birth weight children and a 42% reduction in infections related to neonatal mortality [3, 4] and increases rates of exclusive breastfeeding [5].

The global data shows that coverage of timely initiation of breastfeeding is still below 45% and declining in several countries. In the developing world, only 44% of newborns breastfed within 1 h after delivery [1]. In Latin America and the Caribbean, central and east Europe have lower rates of early initiation of breastfeeding compared to Africa and Asia [4].

There are various factors that influence the timely initiation of breastfeeding. The factors vary from infant to mother’s characteristics and health care provider’s support and encouragement on the benefits of early initiation of breastfeeding [6–10]. To promote early initiation practices, the WHO has developed different interventions. The interventions include infant and young child feeding strategy, baby-friendly hospital initiative, skin to skin contact right after delivery, the practice of rooming-in, delay cord-cutting, early and continuing mother-infant care [11]. Tanzania has adopted the interventions, but the rate of early initiation of breastfeeding is still low. The Tanzania Demographic Health Survey (TDHS) report shows that only 51% of infants are breastfed within 1 h after birth [12].

Some studies have assessed early initiation of breastfeeding at the national level but did not determine the associated factors. Furthermore, existing studies are limited to rural areas such as in the Kilombero and Ulanga districts in the Morogoro region and Rufiji in the Pwani region located in the coastal zone of Tanzania [6]. A previous study in this region reported that 77% of women practiced early initiation of breastfeeding within 1 h after delivery [13]. Still, the study did not assess factors related to the practice. Therefore, we aimed to determine prevalence and factors associated with timely initiation of breastfeeding within 1 h after birth among women with children aged < 24 months in the Kilimanjaro region, northern Tanzania.

## Methods

### Study design and setting

We performed a secondary analysis of data from a cross-sectional study in the Kilimanjaro region, northern Tanzania, conducted by the Institute of Public Health (IPH) of Kilimanjaro Christian Medical University College (KCMUCo). The study aimed to assess the feeding patterns and nutritional status of children under 5 years of age. Data were collected from three districts of the Kilimanjaro region, namely Same and Rombo district councils and Moshi municipal council. The first two districts are rural while the Moshi municipality being urban, hence allowed for rural-urban comparisons.

### Sample size and sampling

**Fig. 1:**
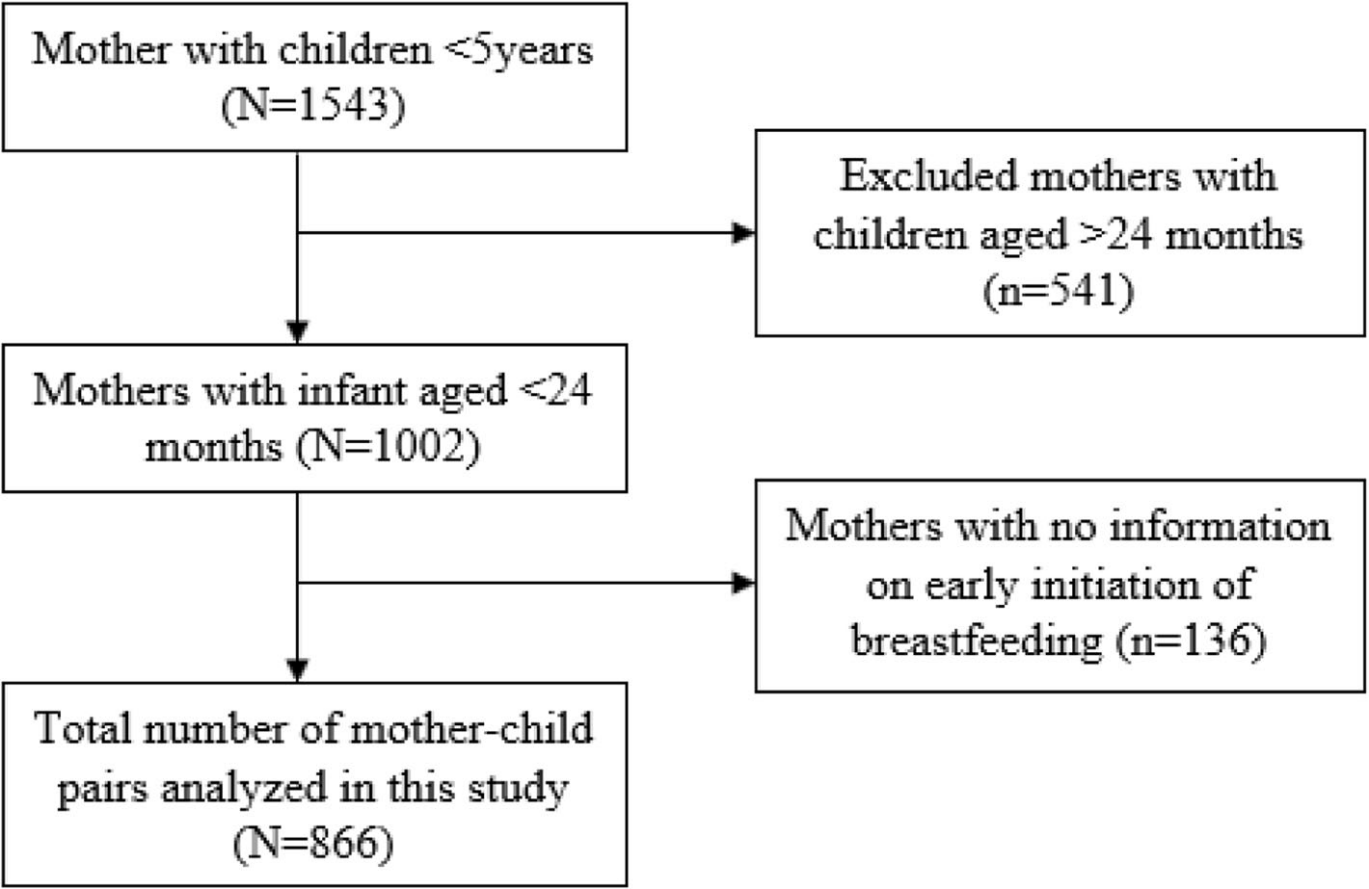
Flowchart of recruitment and inclusion

The parent study enrolled 1543 mothers with children aged less than 5 years and was present on the day of data collection. The study used a multistage sampling technique to select study participants. In this analysis, we excluded 541 observations for children aged 24 months or higher and 136 missing information on early initiation of breastfeeding and analyzed data for 866 mothers with children aged 0–24 months (Fig. 1).

### Data collection methods

The institute of public health at KCMUCo collects data during community engagement activities for years 1–3 medical students. The data are open for any student to access when writing their research projects during year 4 of the medical degree training. Trained doctor of medicine students collected data through face to face interviews using a questionnaire (Additional file 1). The questionnaire collected information on the socio-demographic characteristics of mothers, characteristics of children, reproductive history, and breastfeeding (BF) practices. The questionnaire was in both English and Swahili languages but administered using the Swahili language, a language spoken by all the local people in this setting.

### Study variables and measurements

The dependent variable in this study is the early initiation of breastfeeding among mothers with children less than 2 years. Early initiation of breastfeeding was defined as initiating breastfeeding within 1 h after delivery [1] and assessed by asking mothers, *“How long (in hours) after delivery did you start breastfeeding the child?”.* Mothers who initiated breastfeeding within 1 h were coded as 1 and 0 if otherwise.

Independent variables included socio-demographic characteristics of mothers, particularly age in years, area of residence, tribe, marital status, education level, and occupation. In contrast, children characteristics included age in months, sex, and birth weight, whereby low birth weight consisted of children born with < 2.5 kg. The reproductive history/ characteristics of the mother included the number of times attended ANC coded as < 4 visits or ≥ 4 visits, parity, place of delivery, and whether counseled on breastfeeding during ANC attendance. Breastfeeding practices included colostrum feeding and prelacteal feeds (defined as giving the child anything apart from breast milk after delivery), coded as Yes or No.

### Statistical analysis

Data were cleaned and analyzed using SPSS version 18. We summarized categorical variables using frequencies and percentages, and numeric variables using means and standard deviations. The prevalence of timely initiation of breastfeeding was not rare, i.e., greater than 10%. Therefore, we used the generalized linear models, Poisson family and log-link function estimated the prevalence ratios (PR) with their 95% confidence intervals (CI) for factors associated with timely initiation of breastfeeding instead of classical logistic regression. Variables were significantly associated with timely initiation of breastfeeding if *p*-value< 5%.

## Results

### Respondent characteristics

The mean (SD) age of 866 mothers was 28.3 (SD: 6.8) years, more than two-thirds (36.4%) were aged 30 years or above, and only 4.8% were adolescents (15–19 years). The majority 721 (83.3%) were in union (married or cohabiting with their partners), 702 (81.1%) resided in rural areas, 581 (67.1%) had primary education level, and over half 470 (54.3%) were farmers. Furthermore, the mean (SD) age of children in this study was 12.3 (SD: 7.3) months, and 401 (46.3%) of all (866) children were aged between 13 and 24 months. About half of them were males 439 (50.7), and 110 (12.7%) were born with low birth weight (Table 1).

**Table 1:** Background characteristics of mothers and children (*N* = 866)

Variable	Frequency (n)	Percentage (%)
***Socio-demographic characteristics of mothers***		
**Age (years)**^a^		
15–19	42	4.8
20–24	262	30.3
25–29	243	28.1
30+	315	36.4
**Area of residence**		
Urban	164	18.9
Rural	702	81.1
**Tribe**		
Chagga	401	46.3
Pare	313	36.1
Others	152	17.6
**Marital status**		
In union	721	83.3
Not in union	145	16.7
**Education level**		
Never been to school	37	4.3
Primary education	581	67.1
Secondary education and above	248	28.6
**Occupation**		
Farmers	470	54.3
Business	228	26.3
Others	168	19.4
***Characteristics of children***		
**Age (months)**		
0–5	191	22.1
6–12	274	31.6
13–24	401	46.3
**Sex**		
Male	439	50.7
Female	427	49.3
**Birth weight (Kgs)**		
Low birth weight (< 2.5)	110	12.7
Normal birth weight (≥2.5)	756	87.3
***Reproductive history***		
**Parity**		
One child	262	30.2
Two children	488	56.4
3+ children	116	13.4
**Place of delivery**		
Health facility	767	88.6
Home/others	99	11.4
**Number of times attended ANC**		
< 4 visits	318	36.7
≥ 4 visits	548	63.3
**Counseled on breastfeeding during ANC visits**		
Yes	582	67.2
No	284	32.8
**Child given colostrum**		
Yes	785	90.7
No	81	9.4
**Prelacteal feeds**		
Yes	55	6.4
No	811	93.7

^a^Frequencies and percentages do not tally to the total due to missing values in mother’s age categories

**Fig. 2:**
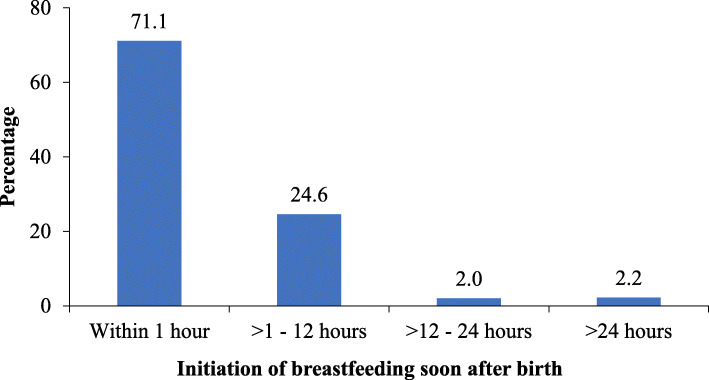
Percentage distribution of initiation of breastfeeding after delivery

The majority of mothers 767 (88.6%) delivered in the health facilities, 488 (56.4%) had two children. About two-thirds 548 (66.3%) attended ≥4 ANC visits during the current pregnancy and 284 (32.8%) of mothers who attended ANC were not counseled on breastfeeding. More than 90 % (90.7%) of all mothers provided colostrum, while about 6.4% gave prelacteal feeds to their infants (Table 2). In this study, the prevalence of timely initiation of breastfeeding within 1 h after birth was 71.1% (Fig. 2).

**Table 2:** Factors associated with timely initiation of BF (*N* = 866)

Variable	N	TIBF n (%)	CPR (95%CI)	***P***-value	APR (95%CI)	***P***-value
***Socio-demographic characteristics of mothers***						
**Age (years)**						
15–19	42	29 (69.1)	0.98 (0.66, 1.44)	0.90	1.07 (0.70, 1.65)	0.75
20–24	262	186 (71.0)	1.00 (0.83, 1.22)	0.98	1.08 (0.83, 1.40)	0.57
25–29	243	175 (72.0)	1.02 (0.83, 1.24)	0.87	1.07 (0.85, 1.34)	0.57
30+	315	223 (70.8)	1.00			
**Area of residence**						
Urban	164	116 (70.7)	0.99 (0.81, 1.22)	0.95	0.99 (0.79,1.24)	0.95
Rural	702	500 (71.2)	1.00			
**Tribe**						
Chagga	401	289 (72.1)	1.03 (0.83, 1.29)	0.77	1.00 (0.79, 1.26)	0.98
Pare	313	221 (70.6)	1.01 (0.80, 1.28)	0.92	1.00 (0.79, 1.27)	0.97
Others	152	106 (69.7)	1.00			
**Marital status**						
In union	721	523 (72.5)	1.13 (0.91, 1.41)	0.27	1.13 (0.90, 1.43)	0.29
Not in union	145	93 (64.1)	1.00			
**Education level**						
Never been to school	37	28 (75.7)	1.08 (0.73, 1.62)	0.69	1.04 (0.66, 1.63)	0.87
Primary education	581	415 (71.4)	1.02 (0.86, 1.22)	0.79	0.97 (0.80, 1.19)	0.80
Secondary education and above	248	173 (69.8)	1.00			
**Occupation**						
Farmers	470	342 (72.8)	1.07 (0.87, 1.33)	0.52	1.06 (0.84, 1.32)	0.64
Business	228	160 (70.2)	1.03 (0.81, 1.31)	0.78	1.02 (0.80, 1.31)	0.85
Others	168	114 (67.9)	1.00			
***Characteristics of children***						
**Age (months)**						
0–5	191	137 (71.7)	1.01 (0.83, 1.24)	0.90	1.02 (0.83, 1.26)	0.83
6–12	274	195 (71.2)	1.00 (0.84, 1.21)	0.96	1.00 (0.84, 1.21)	0.96
13–24	401	284 (70.8)	1.00			
**Sex**						
Male	439	308 (70.2)	0.97 (0.83, 1.14)	0.73	0.97 (0.83, 1.14)	0.71
Female	427	308 (72.1)	1.00			
**Birth weight (Kgs)**						
Low birth weight (< 2.5)	110	75 (68.2	0.95 (0.75, 1.21)	0.70	0.96 (0.75, 1.22)	0.72
Normal birth weight (≥2.5)	756	541 (71.6)	1.00			
***Reproductive history***						
**Parity**						
One child	262	182 (69.5)	1.00			
Two children	488	343 (70.3)	1.01 (0.85, 1.21)	0.90	1.00 (0.80, 1.25)	0.995
3+ children	116	91 (78.5)	1.13 (0.88, 1.45)	0.34	1.15 (0.82, 1.62)	0.42
**Place of delivery**						
Health facility	767	553 (72.1)	0.88 (0.68, 1.15)	0.35	0.91 (0.70, 1.19)	0.49
Home/others	99	63 (63.6)	1.00			
**Number of times attended ANC**						
< 4 visits	318	236 (74.2)	1.00			
≥ 4 visits	548	380 (69.3)	1.07 (0.91, 1.26)	0.41	1.08 (0.91, 1.27)	0.40
**Counseled on breastfeeding during ANC visits**						
Yes	582	423 (72.7)	1.00			
No	284	193 (68.0)	0.94 (0.79, 1.11)	0.44	0.97 (0.81, 1.16)	0.74
**Child given colostrum**						
Yes	785	566 (72.1)	1.00			
No	81	50 (61.7)	0.86 (0.64, 1.14)	0.29	0.88 (0.65, 1.19)	0.41
**Prelacteal feeds**						
Yes	55	18 (32.7)	1.00			
No	811	598 (73.7)	2.25 (1.41, 3.60)	0.001	2.22 (1.38, 3.56)	0.001

*TIBF* Timely initiated breastfeeding (within 1 h after birth); *CPR* Crude odds ratio; *APR* Adjusted Prevalence Ratio

### Factors associated with timely initiation of breastfeeding

There were no statistically significant differences in the prevalence of timely initiation of breastfeeding by respondent characteristics, except for prelacteal feeding. In the unadjusted analysis, mothers who did not give their children prelacteal feeding had a higher prevalence of timely initiation of breastfeeding (PR: 2.25, 95%CI 1.41, 3.60, *p* = 0.001) compared to mothers who provided prelacteal feeds. Although adequate (≥4) ANC visits during present pregnancy were associated with a higher prevalence of timely initiation of breastfeeding (PR: 1.07, 95%CI 0.91, 1.26), this association was not statistically significant (Table 2).

We constructed a multivariable analysis model adjusted for respondent characteristics, including pregnancy history and breastfeeding practices. Adjusted for other factors, not giving children prelacteal feeds remained was significantly associated with a higher prevalence of timely initiation of breastfeeding (PR: 2.22, 95%CI 1.38, 3.56, *p* = 0.001). Again, mothers with ≥4 ANC visits during present pregnancy were more likely to timely initiate breastfeeding (PR: 1.08; 95% CI 0.91, 1.27), but this association was not statistically significant. There was no significant association between other characteristics and the likelihood of timely initiation of breastfeeding in this study (Table 2).

## Discussion

The prevalence of early initiation of breastfeeding was 71.1%. The practice of not giving infants prelacteal feeds was the only factor significantly associated with a higher prevalence of timely initiation of breastfeeding in this study.

According to the WHO classification, the estimate reported in this study is categorized as good [14]. These results indicate that a large proportion of infants gets the benefits of early initiation of breastfeeding, which include protection from infections and improving child survival. Early initiation of breastfeeding helps the child get colostrum, which has immune advantages and protect the child from the risk of infections [15]. However, our estimate is slightly lower than (77%) reported in the previous study done in the Kilimanjaro region [13]. At the national level, only 51% of mothers initiated breastfeeding within the first hour of life [12]. Though the estimated prevalence was good, about 32% of women delayed initiating breastfeeding early in this setting. Therefore, there is a need for encouraging mothers to initiate breastfeeding early due to the essential benefits to the child.

The prevalence of early initiation is higher in South Asian countries [4]. Other countries in Europe and Latin America have the lower practice of early initiation of breastfeeding [4]. In Sub Saharan Africa, Eastern and Southern Africa have better practices of early initiation of breastfeeding, and Ethiopia reported a higher rate (83.1%) [16].

In this study, 90.6% of all mothers gave colostrum to their infant, which is a very positive result. Previous studies in Tanzania and other regions showed that mothers were discarding colostrum for the fear that it is dirty or stale milk [17, 18]. A qualitative study in the Kilimanjaro region reported that mothers give colostrum as they believe it improves child immunity [19]. The finding of the present study indicates that mothers are now aware of the benefits of colostrum feeding.

About 6.4% of mothers in this study gave prelacteal feeds to their infants. The prelacteal feeds given included infant formula milk, water, glucose, and cow’s milk. Not giving infants prelacteal feeds increased the likelihood of timely initiation of breastfeeding in this study. The practice of giving prelacteal feeds is discouraged as it limits the frequency of suckling, and exposes the child to the risk of infections [1, 12, 20]. While ANC attendance during pregnancy was not significantly associated with timely initiation of breastfeeding, there is a need for behavior change interventions and health education, especially during ANC visits to counsel mothers on the risks of giving infants prelacteal. During ANC visits, mothers get the chance to be counseled on proper breastfeeding practices and the benefits to a child’s health. These findings are also similar to studies conducted in India and Ethiopia, which showed that ANC attendance was a predictor for timely initiation of breastfeeding [8, 10, 21].

### Strength and limitation and strength of the study

The strength of this study is that it was community-based and may reflect the early initiation of breastfeeding practice in the Kilimanjaro region. However, this study has some limitations. The prevalence of early initiation of breastfeeding was based on the mother’s ability to remember the practice for the past 2 years. This may lead to recall bias, which is likely to underestimate the prevalence of timely initiation of breastfeeding. Also, our findings may not be generalized to other regions and districts across the country.

## Conclusion

The prevalence of timely initiation of breastfeeding in the Kilimanjaro region was higher than the national estimate. The practice of not giving infants prelacteal feeds increased the likelihood of timely initiation of breastfeeding. There is a need to encourage mothers on the significance of recommended ANC visits and early initiation of breastfeeding to their infants to improve the practice.

## Supplementary information

**Additional file 1.** Questionnaire

## Data Availability

The datasets used and/or analyzed during the current study are available from the corresponding author on reasonable request.

## Abbreviations

ANC: Antenatal Care
AOR: Adjusted Odds Ratio
CI: Confidence Interval
KCMUCo: Kilimanjaro Christian Medical University College
TDHS: Tanzania Demographic and Health Survey
WHO: World Health Organization
