# Supplementary material for: Prevalence and factors associated with timely initiation of breastfeeding in Kilimanjaro region, northern Tanzania: a cross-sectional study
Source: BMC Pregnancy Childbirth. 2020 Sep 1;20:505. doi: 10.1186/s12884-020-03209-y (PMC7465800; doi:10.1186/s12884-020-03209-y)
Supplement: Supplementary file 1 — Additional file 1. Questionnaire [file 12884_2020_3209_MOESM1_ESM.pdf]

QUESTIONNAIRE: INFANT AND YOUNG CHILD FEEDING PRACTICES
Questionnaire Number…………
Circle the answer

**a) ID of participant….**<br>**b) Name of district:**<br>1. Rombo<br>2. Moshi urban<br>3. Same<br>**c) Name of ward:**<br>**…………………**		**d) Name of village/ street:**<br>**.................................**<br>**e) Urban or rural:**<br>1. *urban*<br>*2. rural*<br>**f) Date of last delivery: (dd/mm/yyyy)**<br>**……………**		**g) Age of the last born..........**<br>**h) Tel of interviewee:**<br>**………………………**
**Date of interview:**<br>…………………….		**Name of interviewer:**<br>……………………….		**Verified by:**<br>……………………….
**Maternal socio-demographic and family economic characteristics**				
**1**	Date of birth of woman in years *(DD MM YYYY)*			
**2**	Age of the mother in years		………………………	
**3**	Mother's tribe?		1. Chaga<br>2. Pare<br>3. Sambaa<br>4. Others (specify) …………….	
**4**	Current marital status		1. Married<br>2. Cohabiting<br>3. Single<br>4.Divorced/Separated<br>5. Widow	
**5**	Education level		1. Never been in school<br>2. Primary Education<br>3. Secondary Education (''O'' level)<br>4. Secondary Education (''A'' level)<br>5. Higher learning advanced diploma<br>6. Higher learning degree, masters or PhD<br>7. Others	

**6**	Are you currently employed in work which you received regular month salary?		0. No<br>1. Yes	

**7**	Current occupation of the woman	1. Government employee<br>2. Peasant<br>3. Large scale farmer (> 5 acres)<br>4. Business (< 1 million per month)<br>5. Business (> 1 million per month)<br>6. Enterpreneur<br>7. Others, specify……………..…………………………...…………
**8**	partners education level	1. Never been in school<br>2. Primary Education<br>3. Secondary Education (''O'' or ''A'' level)<br>4. Higher learning advanced diploma<br>5. Higher learning degree, masters or PhD<br>6. Others
**9**	Approximate income **of the woman** per month?	Income in Tanzanian shs ……………………………………….<br><br>*Doesn't have an income write 0*
**10**	What is approximate **family** income per month (yours partner plus your income)?	Tanzanian shs………………………………………………….
**11**	Do you use alcohol?	0. No<br>1. Yes
**12a**	Regarding accommodation, are you living in your own house or rented?	1. Live in our own house<br>2. Living with parents or relatives<br>3. Rented house<br>4. Employers house
**12b**	If you own a house, type?	1. Brick house<br>2. Mud house<br>3. Others…………………………………………………...…………
**12c**	*number of rooms*	
**12d**	If you are renting; are you renting a whole house, some rooms or a room?	1. Rent a whole house<br>2. Rent rooms<br>3. Have rented one room
**12e**	If employers house; number of room?	
**13a**	Toilet facility?	1. No toilet<br>2. Flush toilet<br>3. Pit latrine with slab<br>4. Pit latrine without slab<br>5. Other (specify)………………………………………………………
**13b**	Does the toilet have cement floor?	0.No<br>1.Yes
**13**	Do you share with another family?	0. No<br>1.Yes
**14**	Where do you get your water from?	1. Tap inside the house<br>2. Tap outside the house

		3. Common supply<br>4. Well/river/spring<br>5. Others (specify) ……………………………………………………..……
**15**	Does your house have electricity?	0. No<br>1.Yes
**Food security at household level**		
**16**	How many meals does your household usually have per day including breakfast?	Number of meals…………………………………………………………
**17**	In the past week, on how many days did the household eat meat or fish?	Number of days per week…………………………………………………
**18a**	In the past 12 months have you experienced any food insecurities in your family?	0. No<br>1. Yes
**18b**	How often in the last year did you have problems in satisfying the food needs of the household?	1. Never<br>2. Seldom<br>3. Sometimes<br>4. Often<br>5. Always
**18c**	Name the types of food that were scarce	
**Reproductive Health**		
**19a**	Number of pregnancies	………………………………………………………………
**19b**	Number of times she has ever given birth to?	……………………………………………………………………………
**19c**	How many children are alive?	……………………………………………………………………………
**19d**	How many pregnancies ended with miscarriage/abortion?	……………………………………………………………………………
**19e**	How many pregnancies ended with stillbirth?	……………………………………………………………………………
**19f**	How many children were born alive but died within one month after birth (neonatal)?	……………………………………………………………………………
**19g**	Have you given births to twins?	0. No<br>1. Yes

**Child characteristics**		
**20**	**Date of birth** (dd/mm/yyyy)	

**21**	**Age of the child in months**	
**22**	**Sex of the child**	
**23**	**How many children does a woman have?**	
**24**	**This child named……………………………is your child number???**	
**25a**	How many children under five years (born by a woman and others) who are living in your household?	
**25b**	Number of children aged less than 5 years (< 60 months) a woman has (born by a woman)	
**25c**	Information of children under five in **6a**<br>Child Number<br>DOB (dd,mm,yyyy)<br>Sex (1.male; 2.Female)<br>Alive or dead<br>1…………………………….<br>2…………………................<br>3…………………………….<br>4……………………………..<br>5……………………. ………	
**26**	**Is the child is aged < 24 months (< 2 years)?**	
**27a**	Are you the mother or guardian of this child?	
**27b**	If you are the guardian; how long have the child lived with you (in years)	
**Use of health facilities during pregnancy with child involved in the study**		
**28a**	Did you attend/go for antenatal care/ clinic during your **pregnancy of this baby?**	0. No == *skip to 29*<br>1. Yes
**28b**	How many times did you attend for antenatal care until delivery?	Number of times…………………………………………………
**28c**	During antenatal care visits of **for pregnancy of this baby**, were you told or counseled on breastfeeding issues?	0. No<br>1. Yes<br>*If yes what were you counselled on?*<br>……………………………………………………………………….
**28d**	Which level of care did you get your antenatal care for pregnancy of this child?	1. Dispensary<br>2. Health center<br>*3.* Hospital
**29a**	Where did you give birth of <u>this child</u>?	1.Health facility<br>2. Home<br>3. Others …………………………………….
**29b**	Birth weight of the baby	Kg………………………………………………………………

**29c**	**Before you were discharged,** were you counseled on breastfeeding the newborn?	0. No<br>1. Yes<br>2. Not applicable because delivered at home<br>*if yes what were you counselled on*?............................<br>………………………………………………………………………....
**29d**	Which level of health facility did you deliver your last baby?	1. Dispensary<br>2. Health Center<br>3. Hospital<br>4. Not applicable because delivered at home
**30a**	Did you attend for check up and vaccination of child birth?	0. No<br>1. Yes
**30b**	How frequent did you attend for post-natal/ vaccination care?	1. Once<br>2. Twice<br>3. Three or more
**30c**	Did you receive counseling on breastfeeding or infant feeding when you attended for vaccination services?	0. No<br>1. Yes<br>*If yes what were you counselled on……………………………………………………………………….*

Child Number	DOB (dd,mm,yyyy)	Sex (1.male; 2.Female)	Alive or dead
1			
2			
3			
4			
5			

**Breastfeeding information**		
**31**	How long after delivery did you start breastfeed the baby?	……………………………………………….hours
	So we can summarize the baby started to be breastfed (*interviewer summarize & do not ask the mother)*	1. Within 1 hour after birth<br>2. > 1 hour – 12 hours after birth<br>3. > 12 hours – 24 hours after birth<br>4. > 24 hours after birth
**32**	Was the baby given anything before starting to breastfeed?	0. No<br>1. Yes (*what, mention………………………………………...)*
**33**	Was the baby given colostrum?	0. No<br>1. Yes<br>If no why?
**34**	Is the baby still breastfeeding?	0. No (*if not, why?..)*<br>1. Yes
**35**	Is the child given any other foods apart from breast milk at the moment?	0. No<br>1. Yes
**35**	When did you introduce the following?<br>*water*<br>*cows milk*<br>*light porridge*	At what age?(months)<br>…………………………………………………………..<br>…………………………………………………………..

	Soup<br>*Stiff porridge*<br>Other foods introduced before 6 months?	……………………………………………………………..<br>……………………………………………………………..<br>……………………………………………………………..<br><br>………………………………………………………………
	**Summarize**: Age when the child was given other foods/fluids apart from breast milk *(write the earliest age when the child was introduced to other food or fluids).*	Month..
**Feeding patterns**		
**36**	24 hours recall: can you please tell me fluids/food (child's name……………….) was given yesterday from when she/he waked up until she/he went to sleep?<br>**Write in order from morning to evening**	

**36b**	According to the foods mentioned above (36a) that the child was given, put in which food group the food mentioned fall in to/ or belong (interviewer need to classify the food groups…don't ask this qns)	
**37**	In a typical day, how many servings of fruits does the child receive name(……………….) how many times he/she eats?	
**37b**	In a typical day, how many servings of vegetables? name(……………….) eat or given?	
	**Summarize:** in a typical day how frequent is the child is given fruits and vegetables?<br>(don't ask the mother)	Number of times/day………………………………………………
**38a**	In a typical day, do you prepare special food for (mention name…….) or he /she eats the food prepared for the whole family?	1. Prepare special food<br>2. Child eats family food
**38b**	In a typical day, how frequent do you feed (mention the childs food)**Prompt the mother by starting with what she gives in the morning till sleeping time**	Frequency of feeding per day…………………………………
**39a**	Taking the whole family; what	

	did you eat yesterday from when you woke up till the time you went to bed??<br><br>**Write in order from morning to evening**	

Food group	Types of food mentioned in 36a
Starch/carbohydrates	
Fruits	
Vegetables	
Proteins (except milk)	
Oils and fats	
Milk & milk products	

**39b**	Family food: according to the foods mentioned above (42a) that the family ate, put in which food group the food mentioned fall in to/ or belong (don't ask)	
	**History of infections and treatment**	
40	**Has the child been sick in the past 3 months**	0. No<br>1. Yes<br>If yes what?<br>……………………………………………………………..)
41	**Has the child been admitted in the past 1 year?**	0. No<br>1. Yes<br>If yes what was reason for admission?<br>(………………………………………………………………………………..)
42	**Child has any chronic disease?**	0. No<br>1. Yes mention
43	**Has the child receive any deworming drugs in past 6 months?**	0. No<br>1. Yes

Food group	Types of food mentioned in 39a
Starch	
Fruits	
Vegetables	
Proteins (except milk)	
Oils and fats	
Milk & milk products	

**Influence of customs on breastfeeding and knowledge**		
**44a**	Following customs of this place, what is	

	the child given soon after birth?	
**44b**	According to traditions, what was the child given on the first day out of the house?	
**44c**	Following customs of this place, what is the child given in addition to breast milk in first six months after birth?	
**45**	How long have you exclusive breastfed the baby?	months.........
**46a**	In your understanding how long a woman is advised to breastfeed the baby?	months.........
**46b**	In your understanding how long a woman is advised to breastfeed the baby only breast milk without mixing with other foods or water (exclusive breastfeeding)?	months........
**47**	In your understanding do you think a mother can exclusive breastfeed for 6 months?	0. No<br>1. Yes<br>If no why?<br>…………………………………<br>…………………………………<br>…………..<br>…………………………………<br>…………………………………<br>…………..
**48**	In your understanding do you think there is advantage on mother and child if she practices exclusive breastfeeding for 6 months?	

PHYSICAL AND LAB EXAMINATION FORM

ID NUMBER OF CHILD ………………………………………………

**Mother**	**Variable**	**Value**
	**Height** (Centimetres; cm)	
	**Weight** in (kilograms; kg)	
	Reading 1	
	Reading 2	
**Child Arithropometric**		
**Length or height in Centimeters**		
	Reading 1	
	Reading 2	
**Weight (kg)**		
	Reading 1	
	Reading 2	
	Reading 3	
**Head circumference (Centimeters)**		
**Mid upper arm circumference (cm)**		
**Child blood**		
	**Hemoglobin level (g/dL)**	
	**DBS taken**	1.Yes<br>0.No
IF HEMGLOBIN < 7g/dL REFER TO NEAREST FACILITY FOR CARE		
